# Ethyl cellulose nanodispersions as stabilizers for oil in  water Pickering emulsions

**DOI:** 10.1038/s41598-017-12386-4

**Published:** 2017-09-21

**Authors:** Xia Wu, Li Zhang, Xingzhong Zhang, Ya Zhu, Yuehan Wu, Yan Li, Bin Li, Shilin Liu, Jinping Zhao, Zhaocheng Ma

**Affiliations:** 1 Key Laboratory of Environment Correlative Dietology, Huazhong Agricultural University, Ministry of Education, Wuhan, 430074 China; 2 Jiangsu Province Biomass Energy and Materials Laboratory, Nanjing, 210042 China; 3 Department of Thoracic and Cardiovascular Surgery, Zhongnan Hospital of Wuhan University, Wuhan University, Wuhan, 430071 China

## Abstract

Ethyl cellulose (EC) nanodispersions have been prepared through a facile procedure, a process involved the dissolution of EC into ethanol, followed by dipping it in Xanthan Gum ﻿(XG) solution (0.1%, used as anti-solvent), and then removed the ethanol. The influences of preparation conditions on the structure and properties of the EC nanodispersions were investigated. The prepared EC nanodispersion had a negative surface potential, which contributed to its stabilization. The particle size of the nanodispersions could be controlled by changing the concentration of EC. Furthermore, the EC nanodispersions had a potential application for the stabilization of oil/water Pickering emulsion. The obtained Pickering emulsions showed high stability.

## Introduction

Pickering emulsion is a  new classification of emulsions, in which solid particles are used in place of conventional surfactants^1^. Compared with traditional emulsions, Pickering emulsions have ultra-high stability, low toxicity and environmental responsive character, and thus can be used for drug delivery or as templates for preparation of functional materials. Many types of particles including calcium carbonate^2^, carbon nanotubes^3,4^, PMMA particles^5^, silica nanoparticles^6,7^ were used as stabilizers. Some food systems, e.g. mayonnaise, margarine, whipped cream, are also related to Pickering emulsion system^8^. This type of emulsions could act as a delivery system for active substances, lower calories in food, and modify the texture of food, as well as keep the taste and relish of foods. Some attempts on ﻿the﻿ food-grade particles have been carried out for the use in Pickering stabilization based on natural materials, such as starch granules^9^, chitin nanocrystals^10^, kafirin nanoparticles^11^, whey protein microgel particles^12^, and fat crystals^13^. Moreover, modified biomacromolecules, for example chitosan-tripolyphosphate nanoparticles^14^, and modified starch^15^ were also developed. Pea globulin and a gliadin proteins with gumarabic Gum Arabic^16^ were employed as well. The application of hydrophobic modified cellulose particles for Pickering emulsion has been rarely reported till now. Finding edible, nutrient enriched, low-cost, extensive source and efficient emulsifiers is becoming a hotspot and challenging problem in the food colloids science^17^.We have conducted an intensive research on cellulose polysaccharide^18–20^. Recently we have reported the uniform-sized cellulose colloid particles and the application as stabilizer for Pickering emulsions^21^. It is well known that the surface partial hydrophobic properties of the particles were ideal for the usage in the Pickering emulsions, which is required to ensure the partial wetting by water and oil^22^. In this work, we presented a versatile anti-solvent method to prepare ethyl cellulose (EC) colloidal dispersion. The whole process did not require unnecessary chemical reagents. The EC nanodispersions prepared from various initial raw materials were characterized through measuring the particle size and the zeta-potential. Further investigations were conducted to elucidate the synergistic effect of EC particles and surfactant on the stabilization of oil/water (O/W) emulsions. The influence of the pH, ionic strength, and storage time on the stability of the formed emulsions was also clarified.

## Results and Discussion

### Preparation and properties of EC particles

#### *The influenceof initial EC concentration on the size and charge distribution*

**Figure 1:**
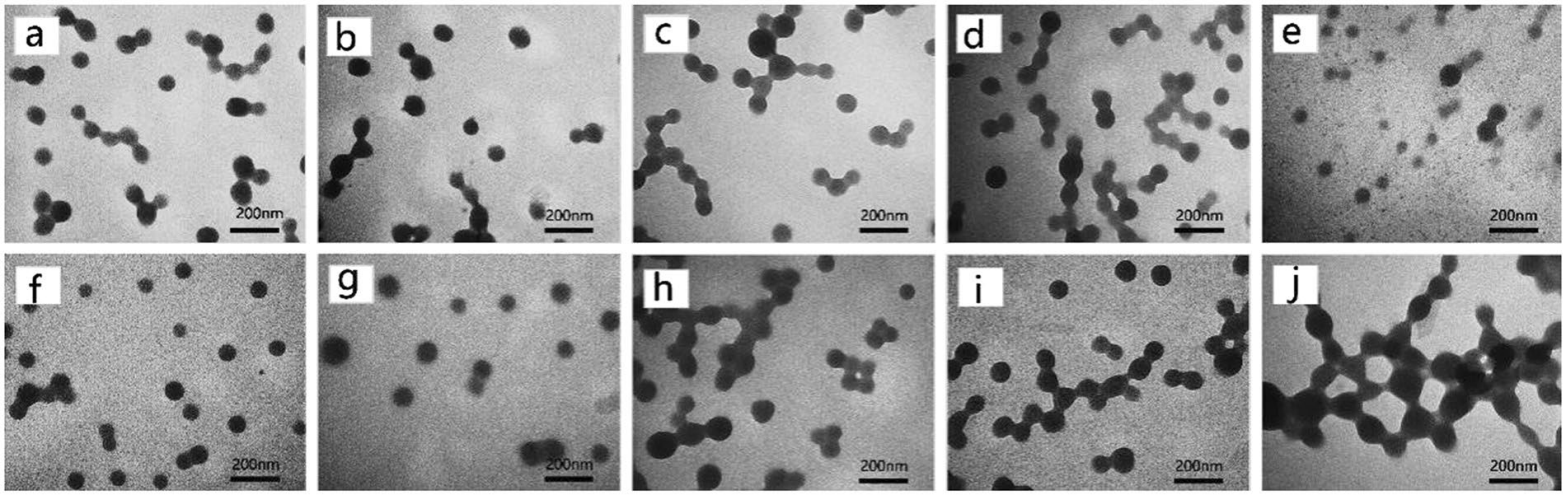
TEM images of the prepared particles with different XG or EC concentrations, (**a**–**d**) and were for the XG with concentration of 0.01%, 0.03%, 0.05% and 0.07%, respectively, and EC solution concentration was 1.0%; (**e**–**j**) were for the EC with concentration of 0.5%, 0.8%, 1.0%, 1.2%, 1.5% and 2%, respectively, XG solution concentration was 0.1%.

The “drowning-out” method was used for the preparation of the nanodispersions from EC polymer materials. The property of the selected continuous phase was important. In this work, ethanol was used to dissolve EC. XG was selected as an interfacial active polymer for the stabilization of the formed solid particles. When the EC solution was added into the continuous phase (water containing XG), ethanol would diffuse into the continuous phase, and this process left EC as the central feature of the resultant particles, and the interfacial active XG on the surface of resultant particles would increase the colloidal stability. Figure 1 showed the TEM images of EC nanodispersions prepared from EC with different concentrations. The results showed that the particle size of the EC nanodispersions was in the range of 60–100 nm. The XG that used as interfacial active polymer had little influence on the particle size of the EC nanodispersions, as shown in Fig. 1a–e and g. Of note, the EC concentration had a slight influence on the particles size of the nanodispersions, e.g., a higher initial concentration of EC led to a little increase in the particle size, as shown in Fig. 1e–j.

**Figure 2:**
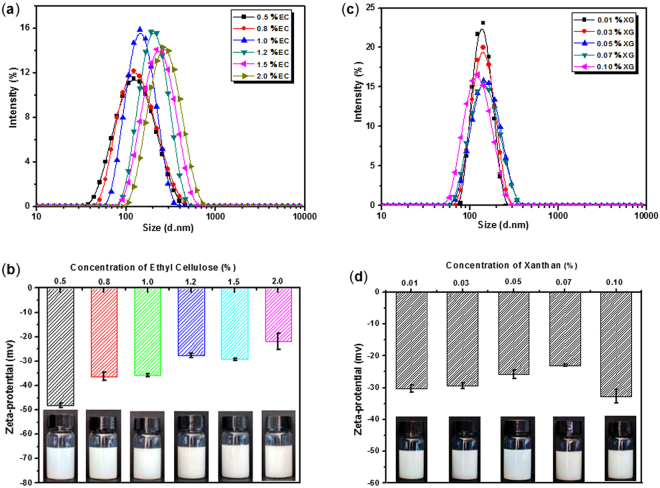
The effect of the concentrations of EC on the particle size distribution (**a**) and Zeta potential (**b**) of the prepared EC nanodispersions, and the concentration of XG solution was fixed at 0.1%. Dependence of particle size (**c**) and zeta-potential (**d**) of EC colloidal particle dispersion prepared from different concentration of the XG, and the concentration of EC solution was controlled to  1.0%.

Figure 2a showed the particle size distribution of the obtained EC nanodispersions. All of the EC nanodispersions prepared from EC solution with different concentrations had a single peak, indicating no aggregation, which was most likely resulted from the electrostatic repulsion between the EC particles. The zeta-potential of original XG solution with concentration of 0.01% was about −48 mV, while the zeta-potential for EC nanodispersions prepared was negative, and it increased dependent on the increase of concentration of the EC solution (Fig. 2b). The zeta-potential of the EC nanodispersions that prepared from EC concentration of 1% solely through the similar conditions was about −29.88 mV (mean particle size ~154.78 nm), and showed a stable state. It was well known that hydrophobic particles would aggregate to form larger masses when they were dispersed in a hydrophilic phase. Looking at the molecular structure of EC, it was difficult to foresee the origin of a native negative charge to ensure the homogeneously disperse of the nanodispersions.

In order to clarify the effects of the XG on the zeta-potential of the EC nanodispersions, the relationship between the XG concentration and the property of the EC nanodispersions prepared from 1% EC was investigated. As it was shown in Fig. 2c,d, the EC nanodispersions prepared from 1.0% EC and different concentration of XG had a single peak, however, the concentration of XG had little influence on the particle size and particle size distribution of the EC nanodispersions, when initial concentration of the EC solution was controlled (Fig. 2c). The EC particles had a stable state in H_2_O, and the zeta-potential  ranged from −30 to −35 mV with the increasing the concentration of the XG solution from 0.01% to 0.1%. The results indicated that the concentration of XG had little influence on the zeta-potential of the EC nanodispersions. Based on our experimental process, the XG would be removed during the dialysis of the EC nanodispersions with deionized water.

#### *The origin of negative charge on surface of EC particles*

FT-IR was used to characterize the molecular structure of the pure XG, EC and achieved EC nanodispersions, as shown in Fig.S1. XG showed a wide peak at 3426 cm^−1^, which was ascribed to the stretching vibration of -OH bonds. Band at 1642 cm^−1^ represented C=O stretching carboxylate group of typical saccharides. A weak peak at 1413 cm^−1^ represented the C-C stretching vibration of the pyranose ring, combined with several peaks between 1048 and 1004 cm^−1^ that denoting the characteristic stretching vibration of C-O-C bond of the anhydroglucose unit^23^. In the FT-IR spectrum of EC, a broad absorption peak appeared at the region of 3550 cm^−1^ was ascribed to the stretching vibration of -OH groups on the EC chains. The bands at 2980 and 2873 cm^−1^ were assigned to the C-H aliphatic stretching position. The absorption peak at 1375 cm^−1^ was ascribed to -CH_3_ bending. These peaks appeared in all FT-IR spectra of the EC nanodispersions. It indicated that it was hard to detect the XG coexisted with the EC nanodispersions after dialysis. It had been reported that OH^−^ could be absorbed on the surface of hydrophobic particles in water^24^, the interfaces of oil-water^25^ when emulsion was stabilized with nonionic surfactants or no surfactants, even at the air-water interface. Such spontaneous absorption of OH^−^ could be occurred on hydrophobic surfaces in contact with water. This hypothesis also could be supported by changing the pH of the EC nanodispersions, where the zeta-potential of the EC nanodispersions would be increased with the decreasing of the pH.

#### *The influenceof pH or ironic strength on stability of EC dispersion*

**Figure 3:**
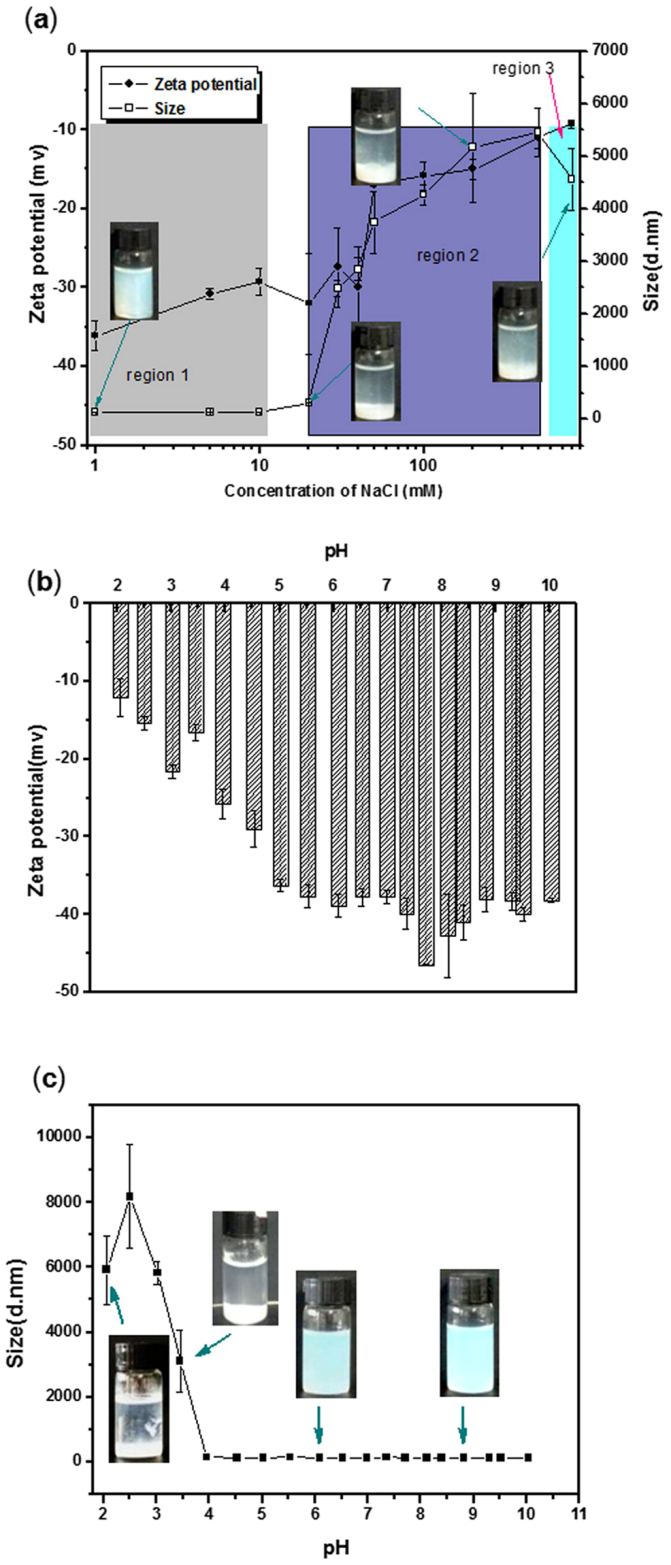
The influence of ionic strength (0–800 mM) on the zeta-potential and particle size of EC dispersion (**a**). The influence of pH on the zeta-potential (**c**) and particle size (**d**) of EC dispersion.

The stability of the EC nanodispersions was characterized by changing the concentrations of added electrolyte NaCl from 1 mM to 0.8 M. Different performances were observed in the EC nanodispersions (Fig. 3a). When the concentration of NaCl was lower than 10 mM, the sedimentation times were almost same to that of without salt. It indicated high stability against agglomeration. This concentration range was noted in the region 1. At the concentration of NaCl in the range of 10–500 mM that noted as region 2, the sedimentation times decreased from about 17 h to 3.0 h when the concentration of the salt was increased. It indicated that the nanodispersions agglomerated significantly, which was comparable to the sedimentation time. The agglomeration contributed to the formation of clusters, leading to a wide range of the sedimentation time. The results suggested that in the region 2 there was a colloidal interaction^26^. When the salt concentration was  increased from 0.5 to 0.8 M, named region 3, a white precipate formed quickly. That indicated that some particles agglomerated rapidly and formed large clusters. In this region, the sedimentation time was about 3 h independent of the salt concentration. The results indicated that agglomeration was occurred faster than sedimentation, it supported that EC nanodispersions had an apparent charge due to OH^−^ ions, which was ionic sensitive.

Figure 3c,d showed the influence of pH on the stability of the EC nanodispersions, when the pH of EC dispersion was decreased from 7.5 to 2.0, the zeta-potential of EC nanodispersions changed from −46 mV to −12 mV, and the mean particle size of EC nanodispersions increased from 130 nm to several micrometers. EC nanodispersions acidified to pH below 3.0 showed phase separation, as the insert photo shown in Fig. 3c. Phase separation was a result of a decreased electrostatic repulsion. The aggregation of the EC particles at either low pH or high salt was a result of a decreased electrostatic repulsion, which was sufficient to destabilize the dispersion. These results supported that there was spontaneous absorption of OH^−^ ion on the surface of EC nanodispersions.

### Hydrodynamic behavior of EC dispersion

**Figure 4:**
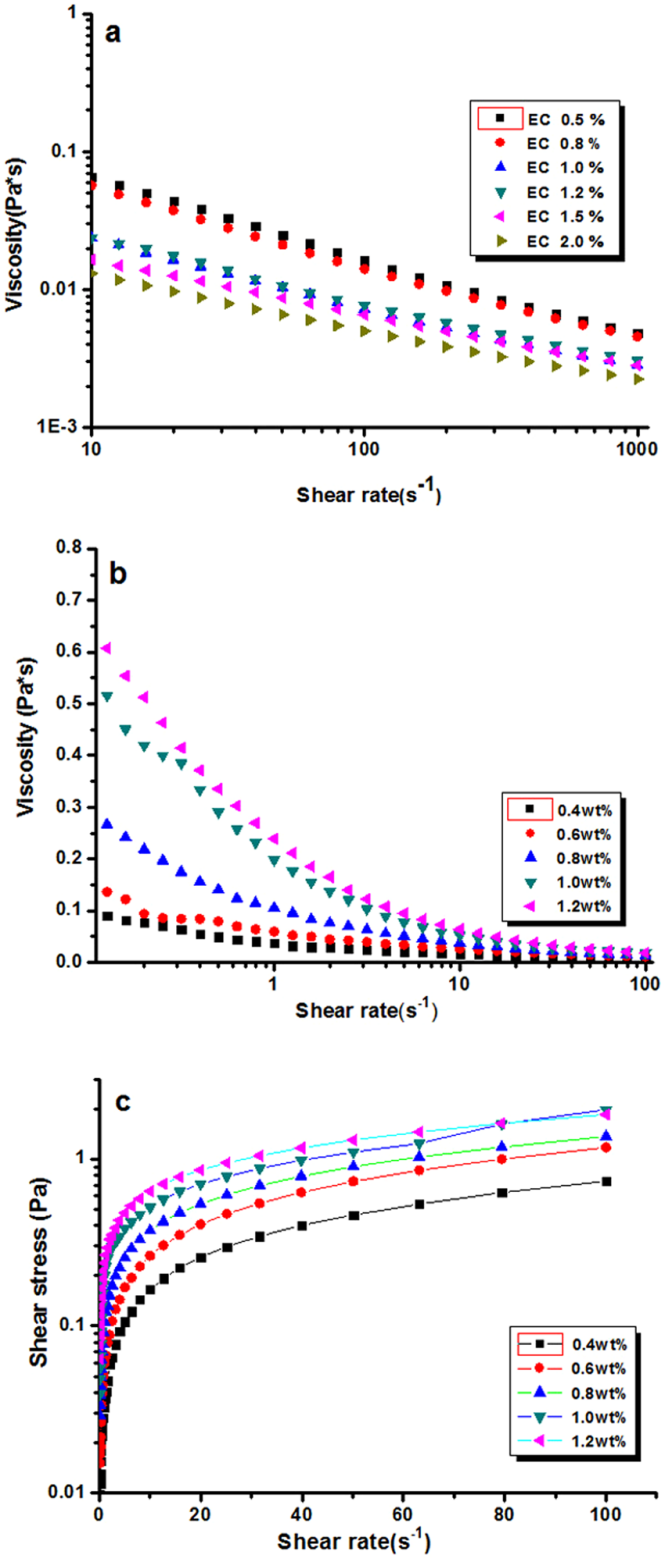
(**a**) Viscosity versus shear rate for EC dispersion prepared with different EC concentrations. (**b**) Plot of viscosity versus shear rate for EC colloidal dispersion with different solid content. (**c**) Plot of shear stress versus shear rate for EC colloidal dispersion with different solid content.

The rheological behavior reflected the viscoelastic properties of nanodispersions under applied shear stress, giving useful information on interactions between adjacent particles in the dispersion, which was technologically important in many applications. Figure 4a demonstrated the plots of viscosity versus shear rate for EC dispersion characterized only by initial EC concentration. EC dispersion was a typical shear thinned fluid since the viscosity decreased with the increase of the shear rate^27^. The viscosity of the dispersion with same solid content was in negative correlation with initial EC solid content. This phenomenon indicated that smaller size particles had relatively thicker solvation layers and volume for fluid dynamics increase^28^. The plot of viscosity versus shear rate for EC dispersion was presented in Fig. 4b. With the increasing of the solid content, the viscosity of the system increased, and each curve exhibited similar profile in the whole process except for the curve of 0.6 wt% and 1.0 wt% at low shear rates. At low shear rates, the dispersion exhibited an obvious non-Newtonian behavior^29^. This shear-thinning behavior became more pronounced at the higher concentration of dispersion due to the stronger interactions between particles with the increasing of concentrations. The viscosity of dispersion tended to reach a Newtonian plateau at high shear rates, viscosity of the system gradually dropped to closer to zero. Figure 4c depicted the plots of stress versus shear rate for nanodispersions with different solid contents. The shear stress increased with the increasing in the solid content of EC dispersion. The Bingham plastic, power-law and Herschel-Bulkey models were applied to fit their shear stress-shear rate curves. The corresponding fit parameters were summarized in Table 1. The power-law and Herschel-Bulkey model were more suitable to the shear stress-shear rate curves compared with Bingham plastic model, which was evidenced by the higher values of *R* ^2^, It could be seen that EC dispersion with higher solid content had increased yield point value, which could be used by food industry to optimize the manufacturing processes of food stuffs.

**Table 1:** Calculated parameters for EC colloidal dispersion with different EC concentrations using Bingham Plastic (BP), Power-Law (PL), and Herschel-Bulkley (HB) Models.

models		Solid concentration of EC dispersion (wt %)				
		0.4	0.6	0.8	1	1.2
BP	τ_0_	0.0489	0.0761	0.1319	0.2010	0.2763
	μ_p_	0.0077	0.0122	0.0140	0.0184	0.0185
	R^2^	0.9653	0.9659	0.9434	0.9530	0.9040
PL	K	0.0370	0.0580	0.1017	0.5374	0.2256
	n	0.6474	0.6507	0.5600	0.5374	0.4512
	R^2^	0.9998	0.9999	0.9997	0.9812	0.9991
HB	τ_0_	0.0022	0.0025	0.0068	0.0778	0.0073
	K	0.0359	0.0567	0.0972	0.096	0.2196
	n	0.6540	0.6555	0.5694	0.6306	0.4566
	R^2^	0.9999	0.9999	0.9998	0.9850	0.9990

### The influence of EC particle content O/W emulsions

**Figure 5:**
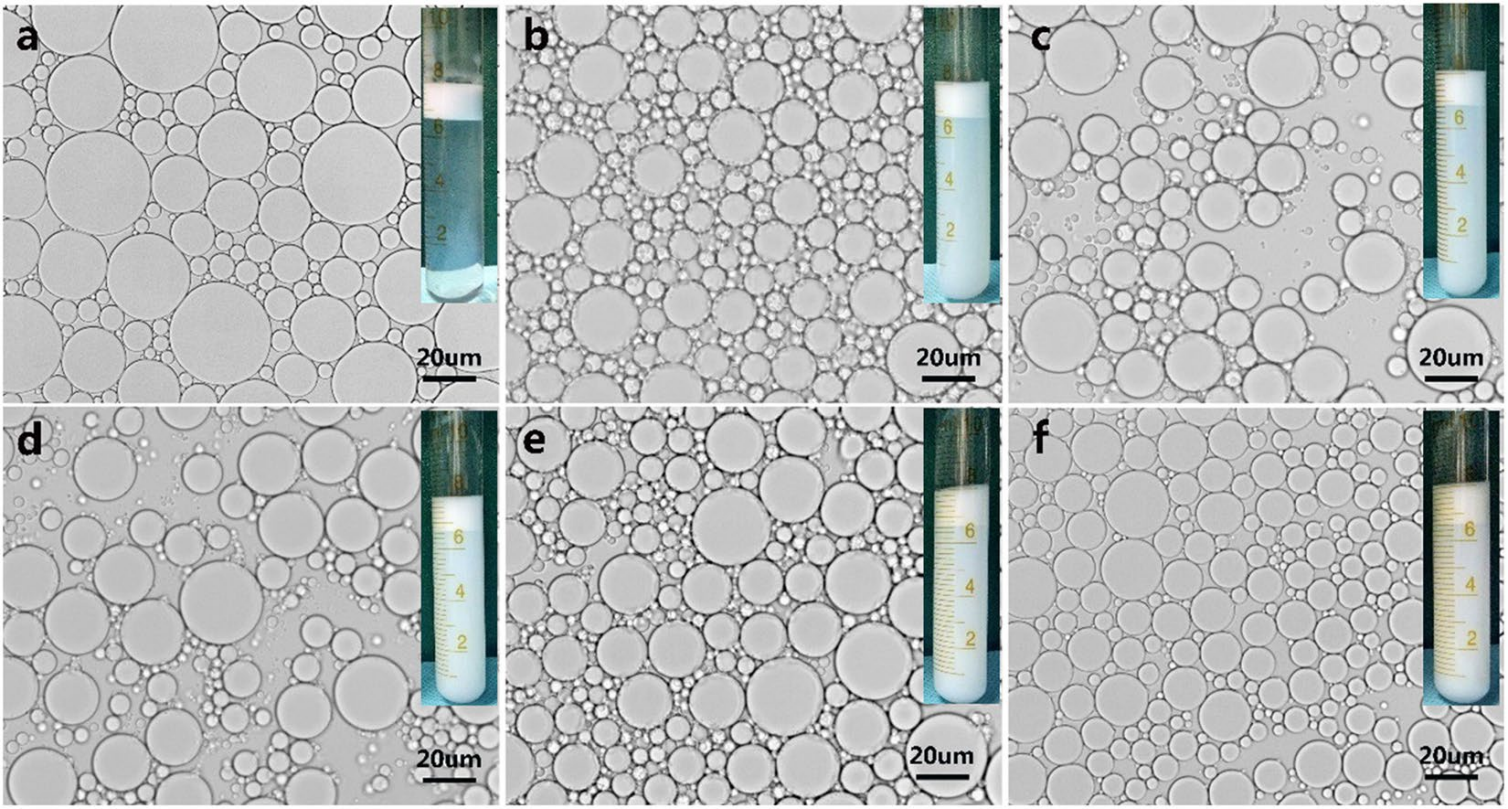
Optical microscopy of Pickering emulsions stabilized by 0.5% Tween 80 in tandem with different concentration of EC dispersion, (**a**) 0%, (**b**) 0.4%, (**c**) 0.6%, (**d**) 0.8%, (**e**) 1.0%, (**f**) 1.2%.

A set of emulsions (the volume ratio of oil phase and water phase was 10:90) stabilized by controlled content of Tween 80 (0.5%) and EC nanodispersions with content ranging from 0 to 1.20% (w/v) were prepared through Homogenization. The results indicated that the emulsion was not stable when it was stabilized only with Tween 80 with low content, and the particle size of the emulsion was not homogeneous, as it was shown in Fig. 5a. In the systematic study, it indicated that the lowest content of EC nanodispersions coexisted with Tween 80 (0.5%) should be set to about 0.40%, otherwise the prepared emulsion was not stable. The highest content of EC nanodispersions was set to 1.20% (w/v), the emulsion remained well flowability. There was little change in the particle size of the emulsions that prepared with the increasing the content of EC nanodispersions, as it was shown in Fig. 5b–f. The size distribution of emulsions shown in Fig.S2 amplify this as well. However, the stability of the emulsion prepared with higher content of EC nanodispersions was increased. In general, a higher content of EC nanodispersions possibly enabled the adsorption of more EC nanoparticles at the oil-water interface, which could reduce the surface tension of the emulsions. It suggested that the EC nanodispersions with a higher content had more powerful ability for the stabilization of the O/W Pickering emulsions. Digital photographs inserted in Fig. 5 were taken after being stored at 4 °C for 12 days, creaming could be observed, this phenomenon often happened in Pickering emulsions, which was ascribed to the oil droplets with a lower density than aqueous phase, but the under-layers of emulsion with higher concentration of EC particle were more turbid. It could be seen that the emulsified phase volume changed hardly with the increasing the content of EC nanodispersions, the opaque bottom water phase suggested the existing of excess EC nanodispersions which did not anchor onto the droplet interface as it was shown in Fig.S3.

**Figure 6:**
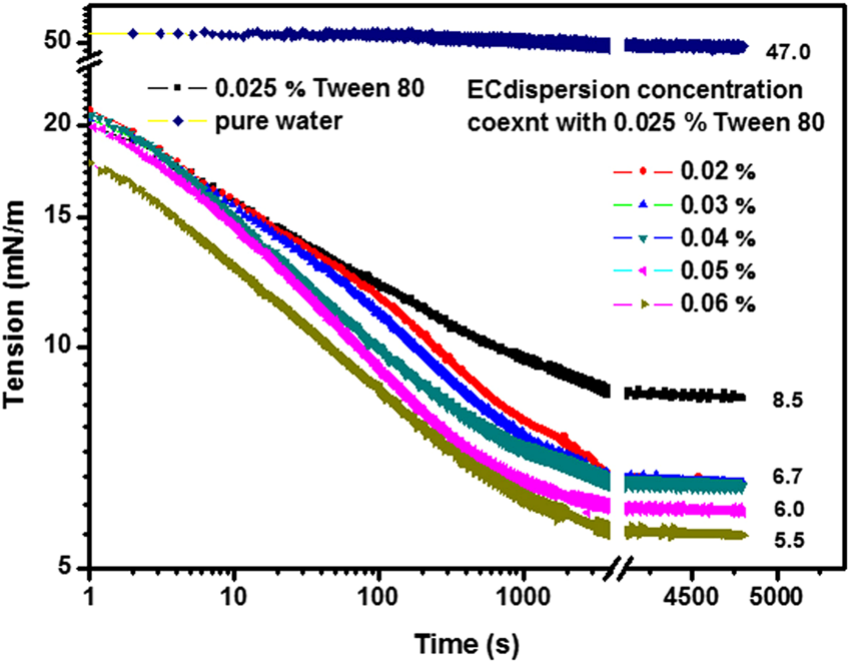
The time-dependence of interfacial tension at the dodecane-water interface on different water phase.

Surface tension measurements at O/W interface were carried out for Tween 80 with different solid content of EC nanodispersions, and it was shown in Fig. 6, the surface tension γ for Tween 80 (0.5%) solution was about 8.5 mN/m, while for the same solution with 0.4% (w/v) EC nanodispersions, it decreased to 6.5 mN/m. Generally, increasing content of EC nanodispersions in the H_2_O containing Tween80 (0.5%) from 0.4% to 1.2% led to a gradual decrease in the residual interfacial tension from 6.5 mN/m to 5.6 mN/m. The shapes of surface tension decreased were similar in the first 100 s, which was mainly attributed to Tween 80 molecules spread to the surface faster than EC nanoparticle. Then the parts of EC nanoparticles diffused to the interface and even displaced some adsorbed tween80 to decrease the contact area of oil–water^30^, thereby reduced the interfacial energy and the interfacial tension decreased faster, and the surface tension decreased slightly. The interfacial tension between pure dodecane and water showed a decrease from 53.3mN/m to about 48.5 mN/m, which could be attributed to the possible surface activity of 1% impurities in dodecane. These observations above all supported that Tween 80 and EC particles do indeed coexist at oil/water interface. The kinetics of adsorption is slowly, with characteristic time of more than 5000 s. Differences were observed in the shape of the isotherm for each concentration of EC dispersion, and it could be seen that with increasing concentration, the decreasing rate of surface tension increased.

**Figure 7:**
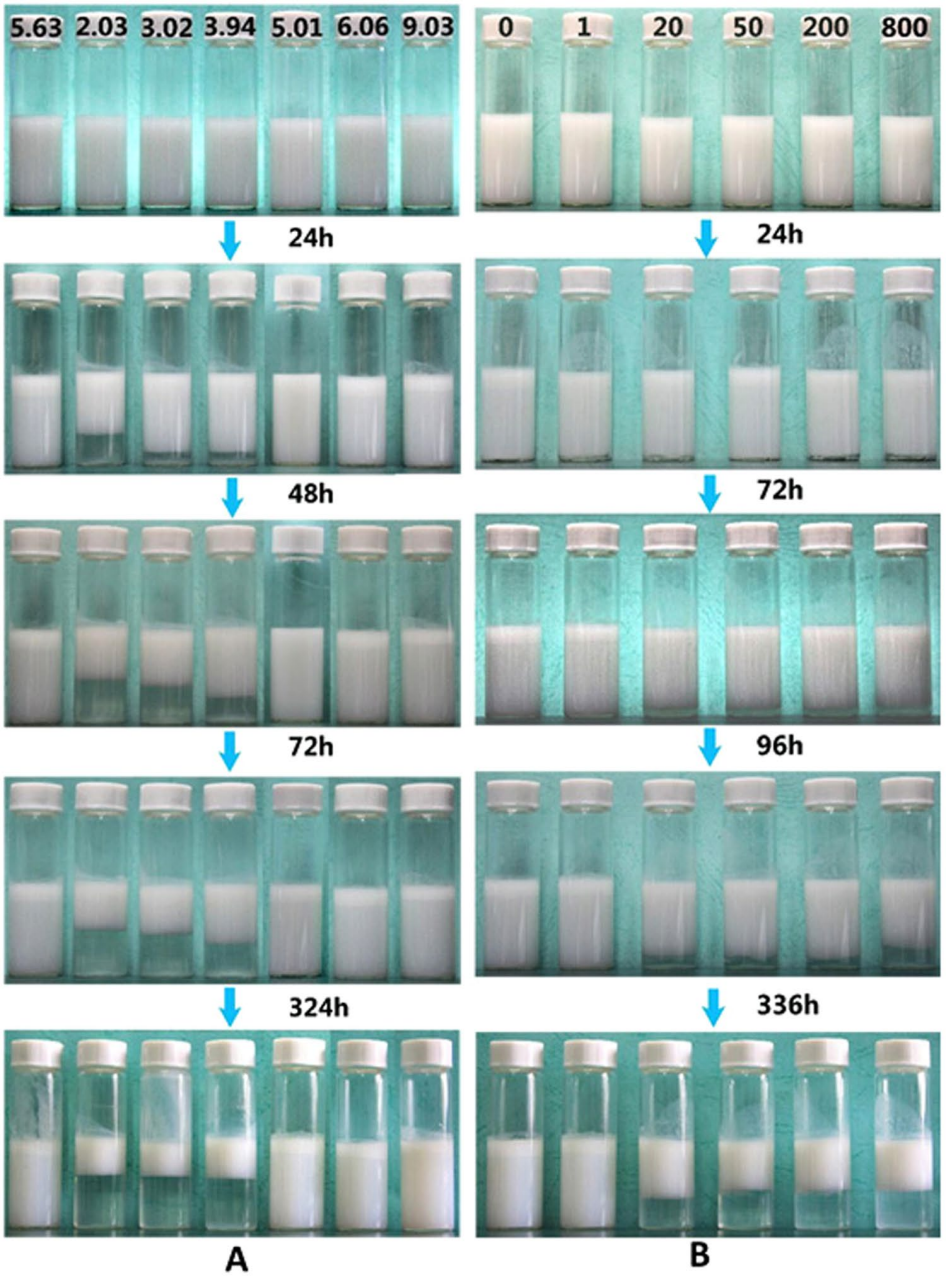
The influence of time on the stability of the Pickering emulsions stabilized by EC nanodispersions under different (**A**) pH or (**B**) ionic strength (concentration of NaCl: mM). The content of EC nanodispersions was controlled to 1.2%.

Since the EC nanodispersions had a negative ζ-potential, the effect of the pH or ionic strength on stability of the emulsions had been investigated, and the data was shown in Fig. 7. It indicated that the O/W emulsions, which were stabilized by EC nanodispersions and Tween 80 (0.5%), had pH sensitive property, and no breaking of the emulsions was observed within range of pH 2.0 to 9.0. However, significant creaming occurred from pH 5.0 to 9.0, which was similar as phenomenon described earlier when the pH of emulsion wasn’t adjusted. It is interesting to note that a stratification with a well-distributed superstratum and a transparent substratum appeared in a pH range 2.0–4.0. The negative charge of particle surface would be screened to a higher value influenced by OH^−^ ion concentration, which would decrease the electrical barrier between particles and the interface agglomeration of particles on the oil droplet surface^31^. Unlike EC dispersion, since the greater difference in density, oil droplets enwrapped with EC particle oil rose to the upper-layer. Furthermore, the speed and degree of condensation were enhanced when emulsion was adjusted to a lower pH. Similar separation of emulsions was shown in Fig. 7B when the concentration of NaCl was higher than 20 mM, which was in consistent with the data shown in Fig. 3. The compression speed of turbid upper layer containing aggregated emulsion droplets and EC particles rose with the ionic strength, and to similar a height at last. Since Tween 80 added in our emulsion was nonionic surfactant, the influence of ionic strength on it could be neglected.

## Conclusions

EC nanodispersions with a mean particle size about 110 nm were prepared, and the particle sizes of the nanodispersions increased slightly with the increasing of the initial concentration of EC solution, nevertheless, there seemed no discernible correlationship between the particle size and XG. The surface charge of the EC nanodispersions could be controlled by adding acid, base or salt, and then its stability was changed. The obtained EC dispersion displayed typical shear-thinning behavior. Moreover, we have shown that oil-in-water emulsion system could be formed by using EC particles in coordination with Tween 80. The emulsion stability of EC nanodispersions was found to be depended greatly on the solid content. The pH and ionic strength could influence the absorption of particles on oil droplets and appearance of emulsion. Since its tunable drop size, decent stability and environmentally responsibility, emulsion stabilized by EC particles owning food grade status may offer a scope for many specific applications in food industry.

## Methods

### Materials

Ethyl cellulose (EC) with an ethyoxyl content of 51% and Xanthan Gum (XG) at USP grade were purchased from Shanghai Aladdin Co., Ltd. Dodecane (99%) was purchased from Shanghai Macklin Biochemical Co., Ltd. Absolute ethanol and Tween 80 were purchased from Sinopharm Chemical Reagent Co., Ltd. Deionized water and Water purified by a Milli-Q system was used for the experiments.

### Preparation of EC nanodispersions

The anti-solvent precipitation method was used to prepare the EC particles. Briefly, EC powder was dissolved in ethanol to obtain the EC solution. XG powder was dissolved in deionized water. Then the EC solution was dropped into the XG solution under mechanical stirring (300 rpm). Vacuum rotatory evaporator and dialysis were employed to remove ethanol and XG separately. Finally, the EC nanodispersions were obtained. To optimize the preparation conditions of EC colloidal particles, the XG concentration was fixed at 0.1%, and the initial concentration of EC solution were set as 0.5%, 0.8%, 1.0%, 1.2%, 1.5% and 2.0%, respectively. A fixed EC concentration was 1.0%, XG concentrations were set as 0.01%, 0.03%, 0.05%, 0.07% and 0.1%.

### Preparation of EC nanodispersions stabilized Pickering emulsion

The ratio of oil to water was set to 1:9 (v:v) and the emulsions were formulated with oil phase: dodecane and aqueous phase which contains (all in weight proportion) 0.5% Tween 80 and various levels of EC colloidal particles (0%, 0.4%, 0.6%, 0.8%. 1.0% and 1.2%). Tween 80 was dissolved in deionized water and then mixed with EC dispersion. Finally the mixture was homogenized (Ultra Turrax T18 digital, Germany) at the speed of 5,000 rpm for 5 min to get the emulsion. In order to investigate the stability of the emulsion, the prepared emulsions (8 mL) were separately transferred into 10 mL glass tubes and stored at 4 °C for 12 days. Photographs were taken after 12 days since the emulsions were prepared.

### Characterizations

The size of the EC particles was characterized with transmission electron microscopy (TEM) (JSM-6390LV, Japan). EC nanodispersions were gently vibrated in a tube before analysis to ensure that they were homogenous, and then one drop of the nanodispersions was added to the surface of the Cu grid (200 mesh), and dried at ambient condition for TEM measurement. The particle size distribution and zeta potential of the nanodispersions were characterized by using a laser diffraction device (MasterSizer2000, Malvern Instruments, Worcestershire, UK). In order to clarify the effect of pH and ionic strength on the stability of the EC nanodispersions, the pH of EC nanodispersions was adjusted by adding either HCl or NaOH to the desired pH value in the range of 2–10. The EC nanodispersions were diluted with salt (NaCl) solutions to several ions concentrations (0–800 mM) and vortexed for 45 s, and the dependence of the particle size and zeta potential of these nanodispersions on pH and ionic strength were investigated. Fourier transform infrared (FT-IR) tests were carried out with a FTIR analyzer (470-Nexus, Nicolet, USA) in the wave number range from 400 to 4000 cm^−1^.The morphology of the Pickering emulsions was imaged by using optical microscopy (Axioskop Zeiss, Germany) equipped with a CCD camera (AxioCam HRC, Zeiss). Particle size and particle size distribution of the emulsions was determined using a laser diffraction device (MasterSizer2000, Malvern Instruments, Worcestershire, UK). Surface tension and interfacial dilational viscoelasticity were characterized through a drop shape tensiometer (Tracker-H, TECLIS, France), the dynamic surface tension was recorded through the axisymmetric drop shape analysis^32^. To ensure enough transparency of samples in cuvette, the water phases for emulsions were diluted 20 times. The stability of emulsions under various pH and ionic strength was evaluated by visual observation; photographs were taken in 14 days after adjusting.

Rheological behaviors of the prepared samples were investigated by using rotational rheometer (Discovery DHR-2, America), the measurement spacing were set to 14μm to record the whole process of shear stress and viscosity. The steady-state shear viscosity was measured at shear rate in ranging from 0 to 1000 s^−1^. All rheological tests were repeated three times with the same sample and performed at temperature of 25 °C unless otherwise mentioned. The colloidal dispersion is a semisolid rather than an actual fluid. While for non-Newtonian fluids, Bingham, Power-Law and Herschel-Bulkey models, they were commonly applied to find the relationship between shear stress and shear rate^33^. The Bingham plastic model was given by the following equation:

1 $${\rm{\tau }}={{\rm{\tau }}}_{0}+{{\rm{\mu }}}_{{\rm{p}}}\,\ast \,{\rm{\gamma }}$$

where τ was the shear stress, τ_0_ was the yield stress, μ_p_ was the plastic viscosity, and γ was the shear rate. From the Bingham plastic model, the yield stress and plastic viscosity could be obtained^34^, while for a more complex fluid, the linear relationship between the shear stress and shear rate no longer exists^35^. Therefore, a developed power-law model was built:

2 $${\rm{\tau }}={\rm{K}}\,\ast \,{{\rm{\gamma }}}^{{\rm{n}}}$$

where K was the flow consistency coefficient, n was the flow behavior index. However, the power-law model was not comprehensive to fit the rheological curves due to the lack of yield point. The Herschel-Bulkey model was developed^36^:

3 $${\rm{\tau }}={{\rm{\tau }}}_{0}+{\rm{K}}\,\ast \,{{\rm{\gamma }}}^{{\rm{n}}}$$

where τ_0_ was the yield stress, K was the flow consistency coefficient, and n was the flow behavior index^37^.

## Electronic supplementary material

Supplementary Information
