# Supplementary material for: Ethyl cellulose nanodispersions as stabilizers for oil in water Pickering emulsions
Source: Sci Rep. 2017 Sep 21;7:12079. doi: 10.1038/s41598-017-12386-4 (PMC5608756; doi:10.1038/s41598-017-12386-4)
Supplement: Supplementary file 1 — Supplementary Information [file 41598_2017_12386_MOESM1_ESM.doc]

**Supplementary information**

**Ethyl cellulose nanodispersions as stabilizers for oil-water Pickering emulsions**

Xia Wu^1,2^, Li Zhang^3^, Xingzhong Zhang^1^, Ya Zhu^1^, Yuehan Wu^1^, Yan Li^1^, Bin Li^1^, Shilin Liu^1,2^, Jinping Zhao^3*^, Zhaocheng Ma1[[1]](#footnote-2)*

1. Key Laboratory of Environment Correlative Dietology, Huazhong Agricultural University, Ministry of Education, Wuhan, 430074, China.
2. Jiangsu Province Biomass Energy and Materials Laboratory, Nanjing 210042, China.
3. Department of Thoracic and Cardiovascular Surgery, Zhongnan Hospital of Wuhan University, Wuhan University,Wuhan 430071, China.


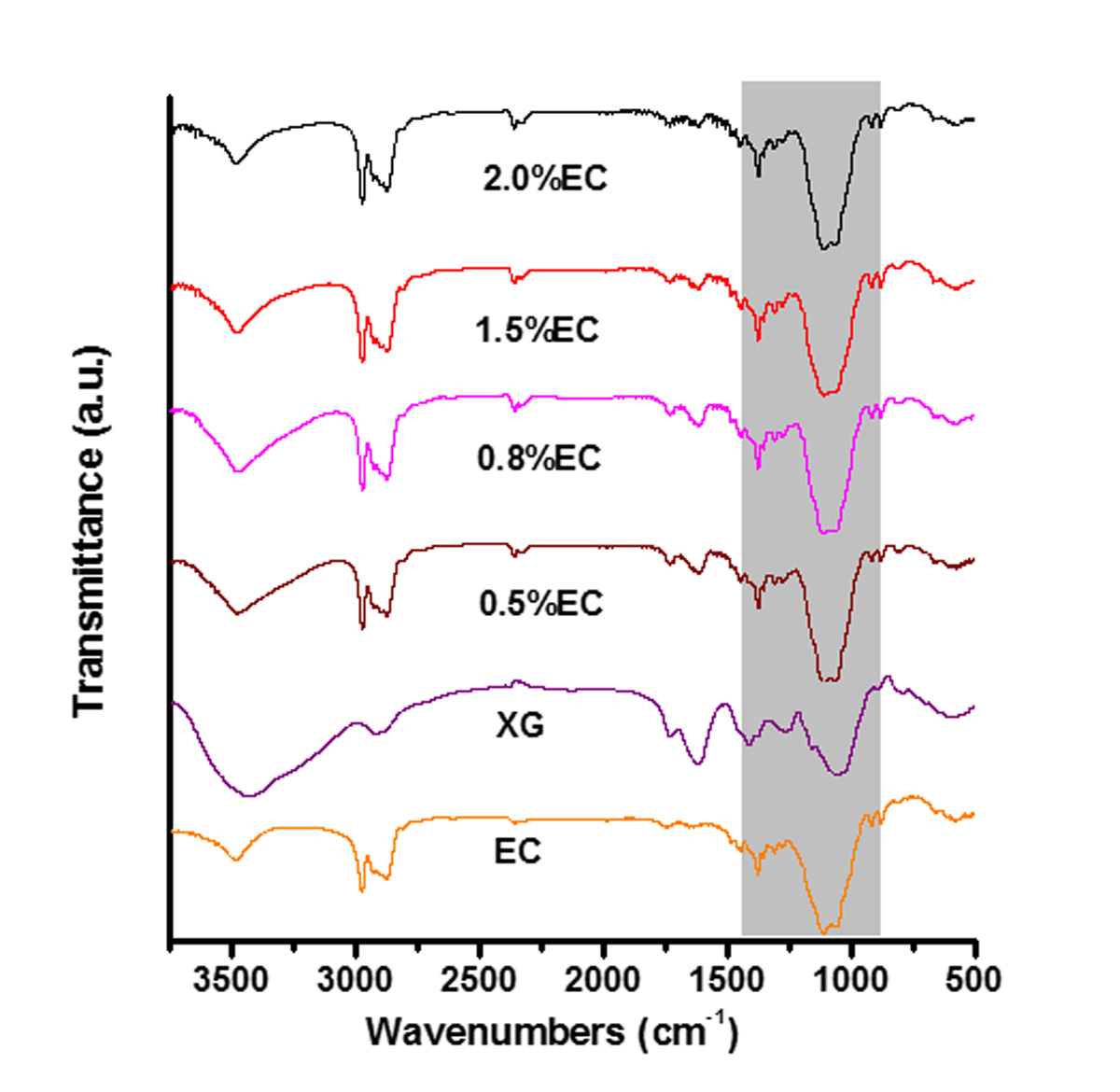


**Fig. S1** FT-IR spectra of ethyl cellulose (EC), xanthan gum (XG) and dried EC dispersions prepared from different concentrations of EC. The concentration of XG is 0.1 %.


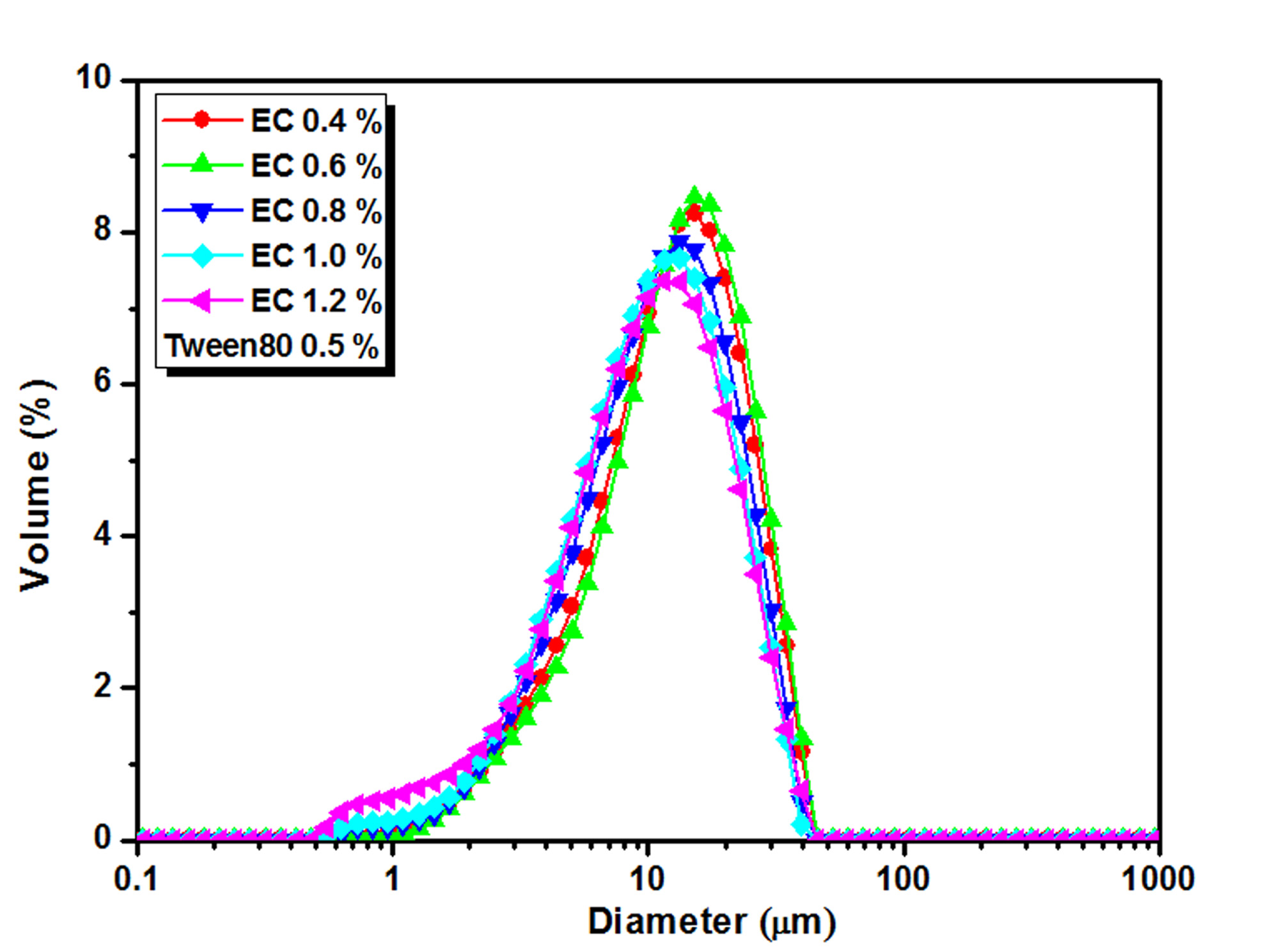


**Fig. S2** Particle size distribution of the emulsions stabilized by EC nanodispersion with different concentrations of EC in 0.5 % Tween 80.


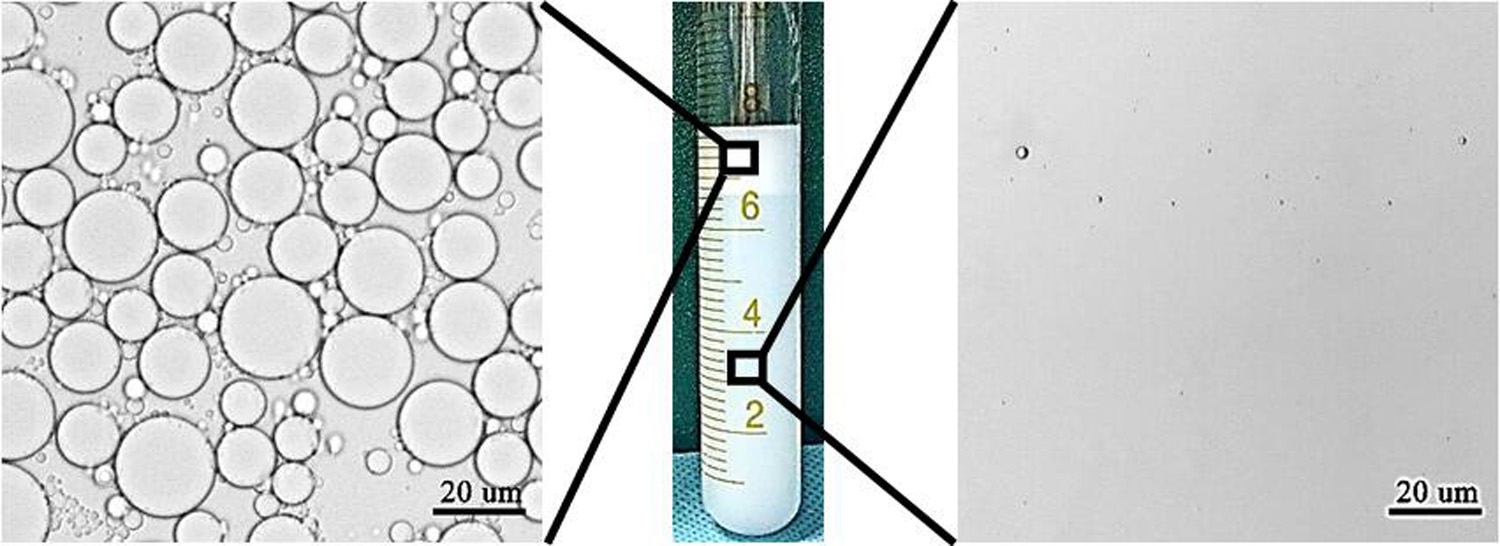


**Fig. S3** Optical images of the emulsion stabilized by 0.8% EC coexisted with 0.5% Tween 80 after being kept for 12 days. Left image, the emulsion section. Right image, the opacified phase.

1. ^*^Corresponding author: [mzhaocheng@mail.hzau.edu.cn](mailto:mzhaocheng@mail.hzau.edu.cn) or [ZhaoJPthx@126.com](mailto:ZhaoJPthx@126.com) [↑](#footnote-ref-2)
